# Research on the impact of the digital economy on the level of industrial structure: An empirical study of 280 cities in China

**DOI:** 10.1371/journal.pone.0298343

**Published:** 2024-03-01

**Authors:** Yanrui Chen

**Affiliations:** College of Finance and Economics, Gansu Agricultural University, Lanzhou, China; Qingdao University, CHINA

## Abstract

As the digital revolution deepens, the digital economy (DE) is reshaping the global industrial structure (IS). This paper utilizes data from 280 Chinese cities between 2007 and 2020 to conduct an in-depth analysis of how DE propels the upgrading of IS and explores the role of human capital (HC) in this process. The research indicates that DE significantly fosters the optimization of IS. Additionally, it was discovered that the growth of HC plays a pivotal mediating role in this evolution, complementing existing research on the relationship between higher education and industrial upgrading. By adopting spatial econometric methods, this study unveils the spillover effects of DE in geographical space, identifying a positive influence on the industrial upgrading of surrounding regions. The paper also confirms the nonlinear characteristics of DE’s impact on IS upgrading, which manifests as a pronounced inverted U-shaped trend, marking a novel discovery. Further findings suggest that in regions with more advanced artificial intelligence technologies, the impact of DE on industrial optimization is more significant, highlighting the role of regional disparities in the digital transformation. In conclusion, the paper proposes policy recommendations based on the research findings to facilitate the development of DE and the elevation of IS levels, thereby promoting high-quality economic growth.

## Introduction

As the DE swiftly advances, digital technology significantly transforms the global economic scenario and the industrial framework. Anchored in information technology and internet infrastructure, DE drives economic growth and innovation via data assets and online platforms. It’s not just a new component of the economic structure but a force that deeply reshapes the overall IS [1, 2]. The application digital technology has not only driven the transformation and upgrading of traditional industries but has also paved the way for the development of new industries and business models [3]. Around the world, numerous nations are ramping up their focus on developing DE, recognizing it as the new driving force for future economic expansion. Digitalization is not only revolutionizing traditional industries but also giving birth to a series of new industries and business models. As digital technology continually advances and becomes more pervasive, the global IS is undergoing unprecedented changes and upgrades. In China, the government is actively advancing DE’s growth, positioning it as a crucial component of the national strategy. The China Internet Development Report 2022 released by the China Academy of Information and Communications Technology in 2022, indicates that amidst the COVID-19 pandemic and global economic fluctuations, China’s DE scaled up to 45.5 trillion yuan in 2021, marking a nominal growth of 16.2% year-on-year. DE has emerged as a key driver in propelling the high-quality advancement of China’s economy [4].

The IS level serves as a critical metric in gauging the economic development of a country or region, reflecting the proportion of different industries in the Gross Domestic Product (GDP). This constantly evolves from traditional to modernized and higher value-added industries, typically accompanying shifts in production methods, technology, human resources, and markets. Existing studies suggest that digital applications, technological innovation, education, human resources, and policies are key drivers elevating IS level [5–7]. For instance, governmental industrial and trade policies can effectively motivate technological innovation, capital investments, and the growth of emerging industries, thereby propelling IS upgrades [8]. However, with the rise of DE, the academia has turned its focus to how the fusion of digital technology and economic growth influences the IS level. Existing literature, approaching from angles such as governmental intervention, circulation efficiency, consumption structure, and technological innovation, demonstrate that DE has a positive impact on enhancing IS level [9, 10]. In the realm of circulation, digital technology permeates various stages from transactions to research, design, and sales, revamping the traditional circulation industry chain, thus enhancing circulation efficiency, which holds significant implications for spurring the secondary industry and benefiting the primary one [11]. Additionally, the ubiquity of digital technology and the evolution of the internet have altered consumer behavior and preferences, massively influencing consumption and ISs [12, 13]. The impact of DE on IS is multi-layered and multi-dimensional, encompassing aspects like IS optimization and upgrade, industry chain reconstruction, and the rise of new industries and business forms.

While existing studies have explored the impact of the DE on IS from multiple perspectives, they often fail to adequately consider the nonlinear effects and spatial spillover effects of DE development. Moreover, there is a notable lack of research on the mediating role of HC in the relationship between the DE and IS. This paper aims to fill this research gap by using rich data from 280 cities in China during 2007–2020 to systematically analyze the path of IS upgrading from the perspective of DE, especially the intermediary role of human capital and the spatial spillover effect of DE development. Our findings reveal that the impact of DE is not only direct but also significantly spatially spillover, which has important implications for regional economic integration and DE development policies. In addition, we innovatively discover a pronounced inverted U-shaped nonlinearity in the effects of DE on IS upgrading, which enriches our understanding of the complex relationship between technological advancement and IS. These contributions offer empirically-based theoretical support for related policies and point out new directions for subsequent research.

Compared to prior research, this paper’s contributions mainly lie in four areas: Firstly, from the novel perspective of DE, it enriches studies on factors influencing the regional IS level. This paper delves into the impact of DE on IS from multiple theoretical perspectives and also empirically substantiates its specific effects, highlighting DE’s potential in facilitating adjustments and upgrades in IS. Secondly, the findings of this paper extend research related to DE development, offering insights for intensifying DE regional collaboration. The analysis in this paper reveals that DE not only enhances IS level of a specific region but also promotes it in neighboring areas, indicating a positive spatial spillover effect, thereby encouraging inter-regional governments to actively foster cross-border collaboration in DE. Thirdly, this paper probes into whether the impact of DE on IS exhibits non-linear characteristics, finding that its positive effect presents certain threshold effects, assisting in understanding more finely the influence of DE on the economic development of regions at different stages and formulating apt policies. Fourthly, by thoroughly examining the role of artificial intelligence technology in enhancing IS, this study establishes a theoretical framework, setting a solid foundation and direction for future research in related domains. This paper underscores the significance of DE in adjusting regional IS, offering empirical evidence to relevant departments for further propelling IS upgrades.

## Method

### Digital economy and level of industrial structure

As digitalization, networking, and intelligence rapidly evolve, DE has increasingly become a major catalyst for economic growth [14]. DE can enhance production efficiency and the efficiency of resource allocation, promoting the transformation of industries towards higher added value, thus profoundly impacting IS [15]. Especially in the manufacturing industry, through the application of technologies like big data, artificial intelligence, and the Internet of Things, production processes can be intelligently managed, making manufacturing more efficient, flexible, and personalized [16], hence promoting the level of IS. Moreover, traditional industrial boundaries are becoming increasingly blurred due to the advancement of digital technology, as digitalization provides opportunities for cross-industry collaboration and integration [17]. This leads to the emergence of new industrial forms, such as the sharing economy, cloud computing, and digital media. These emerging industries cross multiple traditional sectors and redefine the IS [18], enhancing the level of IS. For instance, DE aids the development of the service industry, especially high-added-value service areas like digital content, cloud computing, and e-commerce, resulting in an increased proportion of the service industry within IS [19]. Qi & Chu (2021) indicate that digital technology not only leads to the creation of service industries and products but also integrates extensively with traditional service sectors like finance, healthcare, and education [20]. These are becoming the focal areas for China’s new round of foreign trade expansion. Of course, challenges exist, such as the rapid update of digital technologies, and some traditional industries and enterprises face transformation pressures, with some potentially being phased out. Nevertheless, on the whole, DE continues to promote the level of IS. Therefore, the study proposes the following hypothesis H1.

H1: The DE can promote the level of IS.

### Digital economy, human capital, and level of industrial structure

DE is emerging as a pivotal force in the global economy, impacting the distribution of HC and the transformation of IS [21]. HC, defined as the knowledge, skills, and experience of employees, is now seen as the most valuable resource in the digital age, rather than just physical capital or labor [22]. This implies that, in response to the swift changes in DE, both enterprises and nations must focus on retraining and educating their workforce to ensure employees are equipped to adapt to novel technologies and business models [23], thereby enhancing the level of HC. For example, DE has accelerated the transition from manufacturing-led to digitally serviced-led, and this shift requires higher levels of HC because the digital service and tech industries demand highly skilled labor [24]. Moreover, the level and quality of HC significantly influence the competitiveness and potential for development across different industries [25]. Highly educated and skilled labor can drive technological innovation and the development of knowledge-intensive industries, subsequently altering the pattern of IS [26] and promoting its level. Specifically, a high level of HC can promote the rise of the knowledge economy and push the development of high value-added and high-tech industries [25, 27]. This impact is evident across various countries and cities, with some regions becoming centers for innovation and knowledge-intensive industries due to their high-level talents [26]. Given these insights, this study proposes the following hypothesis H2.

H2: The DE can enhance the level of HC, which in turn promotes the level of IS.

### Discussion on spatial spillover effects

Due to the interconnectedness of geographic spaces and external drivers like policies and regulations, the interaction and connection between industries across different regions are intensifying. Krugman (1991) pointed out that this interconnectedness aligns with the idea of "economic geography" in geographical economics, reflecting the spatial dependence among industries [28]. Porter (1998) stated in "Location, Competition, and Economy" that regional interactions and connectivities play a pivotal role in the growth and innovation of industries, which resonates with the club-convergence characteristics shown by IS level [29]. This clustering means that there’s a "prosper together, decline together" phenomenon between different regional industries, where one region’s economic prosperity might stimulate economic growth in nearby regions, and an economic decline might also be transmitted to other regions. With the development of the regional DE, the emergence and application of new knowledge and production technologies, while improving IS level of the local area, the high mobility of data and online collaboration between platforms also play a huge role. Yang et al. (2018) pointed out in their research that DE is not only changing traditional economic relations between regions but is also promoting the formation of new business models and cooperation relationships [30]. The high mobility of data, as Baldwin (2017) discussed in "The Great Convergence," is redefining boundaries for the global economy [31]. This, in its continuous development, permeates external spaces, gradually elevating IS level of other regions. Considering these points, this study proposes the following hypothesis H3.

H3: The impact of the DE on the IS level has a significant positive spatial spillover effect.

### Examination of non-linear effects

Although this study believes that DE, in general, has a positive impact on the level of IS, its influence is complex and multi-dimensional, with non-linear characteristics. In the initial stages of DE development, the introduction of digital technologies typically results in enhanced production efficiency, which has a direct positive effect on company profits and growth [32]. However, the immature DE in its early stages, constrained by technological bottlenecks, funding restrictions, policy environments, and other external factors, has limited impact on IS level. As Schwab (2017) pointed out in "The Fourth Industrial Revolution," even though digital technology and DE have vast potential, their actual impact in the initial stages is limited by various factors, including the pace of societal paradigm shifts and the challenges of applying technology [33]. As DE continues to evolve, its influence on the level of IS will grow day by day. At this point, due to the continuous permeation of technology, profound transformations appear in industries ranging from finance to manufacturing. Not only do business models and value chains within industries change, but the entire industrial ecosystem is also being reconfigured, with the level of IS continually improving. The influence of DE on the level of IS will not increase indefinitely; its impact will eventually wane again as DE matures. This is primarily because a new equilibrium is reached between the market and technology. Tapscott & Tapscott (2016) believe that as more businesses and organizations gain a deeper understanding and application of DE, the pace of innovation may slow down [34]. This is because most industries have completed their digital transformations, and the level of IS becomes stable. Given these considerations, this study proposes the following hypothesis H4.

H4: The influence of the DE on the level of the IS exhibits an inverted U-shaped characteristic, first increasing and then decreasing.

### Model construction

#### Benchmark model

To measure the influence of DE on IS and confirm the research hypotheses, this study uses data from 280 prefecture-level cities in China spanning 2007 to 2020 as the sample, employing the ensuing fixed-effects model for empirical evaluation. This model controls for static city-specific factors, allowing for a more precise estimation of DE’s impact on IS alterations.


$${is}_{i,t}=\alpha_{0}+\alpha_{1}{de}_{i,t}+\delta X+\gamma_{c}+\omega_{t}+\varepsilon_{i,t}$$ (1)

In Eq (1), where "is" symbolizes the city-level IS, "de" represents the city’s DE development, and "X" indicates various control variables, namely government intervention (peg), population size (ps), degree of openness (ou), economic development level (edl), scale of financial development (fdl), and social consumption (scg). Moreover, γ_c_ represents the fixed effects of the city, ω_t_ stands for the fixed effects over time, and ε_i,t_ signifies the random disturbance term.

#### Spatial Durbin model

Furthermore, to investigate whether there are spatial spillover effects of DE on IS, and to capture the economic connections and potential spillover effects between regions, we introduced the following spatial Durbin model. This model takes into account interactions in space, allowing for the level of IS in one city to be influenced by DE levels of neighboring cities, which aligns with the reality of regional economic integration and network effects.


$${is}_{it}=\alpha_{0}+\alpha_{1}{de}_{it}+\beta_{1}\sum_{j}w_{ij}{de}_{it}+\alpha_{2}X+\beta_{2}\sum_{j}w_{ij}X+\rho \sum_{j}w_{ij}{ic}_{it}+\gamma_{c}+\omega_{t}+\varepsilon_{it}$$ (2)

In Eq (2), where "ρ" denotes the spatial lag auto-regressive coefficient, and "w_ij_" represents the element of the spatial weight matrix.

#### Mediating effect model

We further investigate the role of HC as a mediating variable. Following the three-step mediation effect model by Wen and Ye (2014) [35], this study constructs the subsequent models to inspect the role of HC in DE affecting IS:

$${is}_{i,t}=\alpha_{0}+\alpha_{1}{de}_{i,t}+\delta X+\gamma_{c}+\omega_{t}+\varepsilon_{i,t}$$ (3)

$${hc}_{i,t}=\alpha_{0}+\alpha_{1}{de}_{i,t}+\delta X+\gamma_{c}+\omega_{t}+\varepsilon_{i,t}$$ (4)

$${is}_{i,t}=\alpha_{0}+\alpha_{1}{de}_{i,t}+\alpha_{2}{hc}_{i,t}+\delta X+\gamma_{c}+\omega_{t}+\varepsilon_{i,t}$$ (5)

In Eqs (3)–(5), "hc" signifies the city’s HC level, and Eq (3) aligns with (1).

#### Threshold model

To further assess if the promotional effect of the digital economic development on IS manifests non-linear characteristics, the ensuing panel threshold model is formulated. This model can reveal whether the intensity of influence on IS upgrading will change under different stages of DE development level. Through such a model setting, we can not only examine the overall effect of DE, but also analyze its differentiated impact at different levels of development, providing a more detailed basis for policy making.


$${is}_{i,t}=\alpha_{0}+\delta_{1}{de}_{it}*I(de\leq \gamma_{1})+\delta_{2}{de}_{it}*I(\gamma_{1}<de\leq \gamma_{2})+\delta_{3}{de}_{it}*I(de>\gamma_{2})+\delta X+\gamma_{c}+\omega_{t}+\varepsilon_{i,t}$$ (6)

In Eq (6), where I(·) is the indicator function. Upon threshold testing, a double-threshold model is selected, with γ_1_ and γ_2_ as the threshold values. Specifically, when "de" is less than γ_1_, the influence coefficient of DE on IS is δ_1_. When "de" is between γ_1_ and γ_2_, the influence coefficient is δ_2_, and when "de" surpasses γ_2_, the coefficient is δ_3_.

### Variable definition

#### Core explanatory variable: Digital economy

Digital Economy (de). Since current literature lacks a unified measurement approach for the city-level DE, referencing the studies of Wei et al. (2022) [36], this research integrates the number of broadband internet users per hundred, the proportion of software industry employment in urban units, per capita telecom service volume, and mobile phone users per hundred. These indicators are standardized, dimensionally reduced through principal component analysis, and synthesized into a DE index.

#### Dependent variable: Industrial structure

Various methods measure the level of IS, such as the structural hierarchy coefficient method, cosine angle method, and the ratio of the tertiary to the secondary industry. Given the industrial development trajectory, where the share of agriculture diminishes with industry’s growth and non-agricultural and service orientations dominate, this research, inspired by Fu (2022) [37], utilizes the tertiary to secondary industry ratio as the metric for IS. A higher ratio indicates a more advanced structure.

#### Mediating variable: Human capital

To delve deeper into the mechanisms through which DE fosters the enhancement of IS levels, this paper treats HC as a mediating variable. Education and learning are generally the main pathways to HC improvement. Drawing on the research by Zhan & Liu (2020) [38], this paper measures the level of urban HC using the proportion of students in general higher education. This ratio can be considered a comprehensive indicator, as it not only reflects the current state of HC but also signifies an important aspect of a city’s potential for future development. Specifically, the paper utilizes the ratio of students in regular higher education institutions to the overall city population as a metric for the level of urban HC.

#### Control variables

Building upon prior research [3, 6, 37] and aiming to minimize biases from omitted variables, this study introduces control variables: government intervention (peg), population size (ps), openness degree (ou), economic development level (edl), scale of financial development (fdl), and social consumption (scg). Specifically, government intervention is quantified by local fiscal expenditures to GDP ratio; population size by the log value of the city’s permanent residents; openness by the utilized foreign investment amount relative to that year’s GDP; economic development by the city’s GDP log value; financial development by the year-end loan balance of financial institutions relative to GDP; and social consumption by the residents’ consumption to GDP ratio. The principal variable definitions are demonstrated in Table 1.

**Table 1:** Variable definition table.

	Variables	symbols	Definitions
Dependent Variable	Industrial structure	is	Output value of tertiary industry/output value of secondary industry
Core Explanatory Variable	Digital economy	de	It was synthesized by principal component analysis
Macro level control variables	Government intervention	peg	Expenditure within the general budget of local finance/Gross regional product
	Population size	ps	The logarithm of the city’s permanent population
	External development degree	ou	Amount of foreign capital actually utilized by the city/gross regional product
	Level of economic development	edl	The logarithmic value of the gross urban product
	Financial development level	fdl	City year-end financial institution loan balance/gross regional product
	Social consumption	scg	Household consumption/Gross regional Product

#### Data description

This research undertakes an empirical analysis using panel data from 280 prefecture-level Chinese cities spanning the years 2007 to 2020. Variables with very few missing values have been interpolated using a linear interpolation method, resulting in a balanced panel data of 3,920 samples. Furthermore, the principal data sources include the China City Statistical Yearbook, Wind database, China Research Data Service Platform (CNRDS) database, along with various city statistical yearbooks and bulletins across different years. Table 2 below presents the descriptive statistics of the key variables.

**Table 2:** Descriptive statistics.

Variables	Obs	Mean	SD	Median	Min	Max
is	3920	0.9718	0.5520	0.847	0.094	5.350
de	3920	0.1594	1.1606	-0.139	-1.130	12.571
peg	3920	0.1876	0.1409	0.159	-0.256	2.853
ps	3920	5.8690	0.6990	5.889	3.125	8.074
ou	3920	0.0189	0.0199	0.013	0.000	0.215
edl	3920	16.3883	0.9876	16.335	13.335	19.774
fdl	3920	0.9375	0.6125	0.760	0.075	9.622
scg	3920	0.3674	0.1369	0.357	-1.555	1.505

## Results

### Benchmark regression results

The regression results for the baseline model of this study are presented in Column (1) of Table 3. The coefficient of DE is 0.0293, indicating a positive effect of DE on the level of IS, significant at the 5% level. For every unit increase in DE, IS level will rise by 0.0293 units. As DE advances, the level of IS continues to elevate, reinforcing the derivations made in our theoretical analysis. The evolution of digital technology has given rise to knowledge-intensive industries such as artificial intelligence, the internet of things, and cloud computing, steering traditional industries toward digital and high-end new types of industries, thereby boosting operational efficiency and prompting the upgrade of IS. The empirical results of this study indicate that DE positively fosters the enhancement of IS levels, which is consistent with modern growth theories. Particularly, the endogenous growth theory underscores the pivotal importance of knowledge innovation and technological progression in driving economic growth [39]. The development of DE, particularly the widespread application of Information and Communication Technologies (ICT), has facilitated the accumulation of knowledge and accelerated innovation activities, thereby driving the optimization of IS. For instance, the expansion of the service sector and the evolution of manufacturing have partly gained from the incorporation and utilization of digital technologies, which have made production processes more streamlined and diversified the range of products and services.

**Table 3:** Benchmark regression results.

	(1)	(2)	(3)	(4)
Variables	is	is	is	is
de	0.0293**		0.0271*	0.2907**
	(0.0145)		(0.0158)	(0.1389)
Lde		0.0252*		
		(0.0146)		
Control	YES	YES	YES	YES
City_FE	YES	YES	YES	YES
Year_FE	YES	YES	YES	YES
Obs	3920	3640	3500	3920
R^2^	0.8782	0.8783	0.8458	

Note: *, ** and *** passed the significance test at the level of 10%, 5% and 1% respectively, the same below.

The empirical analysis of the relationship between DE and industrial upgrading in this study is not only statistically significant but also theoretically profound. Our findings enrich the endogenous growth theory by clarifying, in the context of the modern economy, how DE acts as a new type of ’general-purpose technology’ that promotes industry structure optimization by fostering innovation and technological diffusion. Our research fills the gap in the current literature regarding the impact of DE on the growth of the service sector and the transformation of manufacturing, providing a fresh perspective to understand the changes in economic structure under the backdrop of the fourth industrial revolution.

### Robustness tests

#### Lagged treatment

To verify the stability of the outcomes, in line with prior research, the primary explanatory variables and control variables are set one period behind, leading to Lde, Lpeg, Lfdl, Lscg, Lps, Lou, and Ledl. As illustrated in Column (2) of Table 3, the coefficient of Lde is 0.0252, significant at the 10% level. This continues to demonstrate that DE fosters an elevation in IS levels, aligning with previous findings.

#### Excluding direct-administrated cities and provincial capitals

Further, to eliminate potential biases from differing city administrative structures, the sample excluding direct-administrated cities and provincial capitals is re-analyzed. As displayed in Column (3) of Table 3, the coefficient of DE is 0.0271, significant at the 10% level. This also signifies that DE fosters an uplift in IS level, confirming the robustness of our findings.

#### Instrumental variable regression

Given the potential reverse causality between the growth of DE and the optimization of IS, combined with multiple factors affecting IS, we employ the instrumental variable approach to alleviate endogeneity concerns. Following the approach of Zhang et al. (2020) [40], this research adopts the spherical distance from a city’s location to Hangzhou as the instrumental variable for DE. The birthplace of digital finance epitomized by Alipay is Hangzhou, which logically should be advanced in DE. Therefore, cities closer to Hangzhou geographically should exhibit a more developed DE, meeting the relevance requirement for the instrument. Additionally, as geographical distance is exogenously determined, it meets the exclusivity criterion. The outcomes of the second-stage regression are displayed in Column (4) of Table 3, where DE’s coefficient is 0.2907, significant at the 5% level. This reaffirms that DE consistently promotes an upgrade in IS levels, even after accounting for potential endogeneity. Furthermore, the Wald F statistic is 33.232, passing the weak instrument test.

#### Examination of spatial spillover effects

Prior discussions elucidated the driving effect of DE on the level of IS. Existing literature suggests spatial spillover effects in the influence of the internet and DE on regional economic development [41]. This section endeavors to understand the influence of DE on IS upgrading from the perspective of spatial spillover effects. For robustness, spatial economic analyses are conducted using spatial geographical distance matrix, spatial economic distance matrix ($W_{ab}=\frac{1}{\left|\bar{Q_{a}}-\bar{Q_{b}}\right|},\;\bar{Q_{a}}\;and\;\bar{Q_{b}}$ are the average GDP per capita of city a and City b during 2003–2020, respectively), and spatial economic-geographic nested matrix. Initially, a spatial autocorrelation analysis is performed on DE and IS. Due to space constraints, only the results from the spatial geographical distance matrix analysis are reported. The global spatial autocorrelation index is presented in Table 4. Both Moran’s I values for DE and IS are significantly positive, indicating spatial autocorrelation, suggesting agglomeration in their spatial distribution.

**Table 4:** Moran’s I values.

	de			is		
year	Moran’s I Value	Z Value	P Value	Moran’s I Value	Z Value	P Value
2007	0.0939	21.8387	0.0000	0.0164	3.8841	0.0001
2008	0.0930	21.3561	0.0000	0.0129	3.2190	0.0013
2009	0.0826	18.8529	0.0000	0.0181	4.2366	0.0000
2010	0.0667	14.6305	0.0000	0.0153	3.7025	0.0002
2011	0.0840	17.8550	0.0000	0.0150	3.6859	0.0002
2012	0.0851	18.0855	0.0000	0.0145	3.5790	0.0003
2013	0.0765	15.9805	0.0000	0.0146	3.5960	0.0003
2014	0.0801	16.7742	0.0000	0.0212	4.9087	0.0000
2015	0.0773	16.0738	0.0000	0.0244	5.5195	0.0000
2016	0.0715	14.8208	0.0000	0.0302	6.6429	0.0000
2017	0.0788	16.2594	0.0000	0.0317	6.9389	0.0000
2018	0.0765	15.6590	0.0000	0.0328	7.1363	0.0000
2019	0.0518	10.8485	0.0000	0.0420	8.8474	0.0000
2020	0.0449	9.4437	0.0000	0.0390	8.2807	0.0000

Subsequently, drawing from Elhorst (2014) [42], the spatial Durbin model is adopted to re-examine the influence of DE on IS. The results are displayed in Table 5, where Columns (1), (2), and (3) correspond to regressions using the spatial geographical distance matrix, spatial economic distance matrix, and spatial economic-geographic nested matrix, respectively. In all cases, the coefficients of de and W*de are both positive and significant, confirming the positive influence of DE on IS level, even when accounting for spatial factors. Furthermore, the indirect effects, which can be seen as spillover effects, are positive under all three matrices, further testifying to DE’s positive influence on neighboring cities’ IS level. This study finds that the impact of DE on IS has spatial spillover effects, which can be understood through the lens of new economic geography [43]. The expansion of DE not only enhances IS of the local area but also affects the industrial development of neighboring areas through network effects and market connections. This type of inter-regional interaction and synergy has been widely discussed in economic geography and is corroborated in the empirical analysis of this paper.

**Table 5:** Spatial regression results.

	(1)	(2)	(3)
Variables	is	is	is
de	0.0268***	0.0305***	0.0295***
	(0.0066)	(0.0066)	(0.0066)
W*de	0.2767***	0.0303*	0.0533***
	(0.0989)	(0.0182)	(0.0201)
de (Direct)	0.0303***	0.0310***	0.0307***
	(0.0068)	(0.0067)	(0.0067)
de (Indirect)	0.9995**	0.0332*	0.0629***
	(0.4538)	(0.0176)	(0.0202)
de (Total)	1.0298**	0.0641***	0.0936***
	(0.4555)	(0.0197)	(0.0223)
Control	YES	YES	YES
City_FE	YES	YES	YES
Year_FE	YES	YES	YES
Obs	3920	3920	3920
R^2^	0.0050	0.0015	0.0062

The discovery of spatial spillover effects further expands the research scope of new economic geography, providing empirical support for how DE promotes regional IS upgrading through inter-regional network effects and market connections. This aspect is not commonly found in previous research. Our results emphasize the importance of inter-regional synergy and agglomeration forces in economic development, particularly in the era of globalization and digitization.

#### Mediation effect test

To determine whether DE’s advancement can uplift the HC level, thereby affecting IS level, regression is conducted following the mediation effect model. The findings from the three-step regression are shown in Columns (1), (2), and (3) of Table 6. In Column (2), the coefficient for DE is 0.0004, significant at the 1% level. This denotes that DE does enhance the HC level. In Column (3), the coefficients for DE and ’hc’ are 0.0319 and 2.6585, respectively, both significant at the 1% level, indicating the positive influence of HC level uplifted by DE on the upgrade of IS. The mediation effect test validates that DE does boost the HC level, subsequently promoting IS upgrade. This is in line with Romer’s theory that technology and HC combine to promote economic growth.

**Table 6:** Mediation effect regression results.

	(1)	(2)	(3)
Variables	is	is	is
de	0.0293***	0.0004***	0.0319***
	(0.0069)	(0.0001)	(0.0070)
hc			2.6585***
			(0.8411)
Control	YES	YES	YES
City_FE	YES	YES	YES
Year_FE	YES	YES	YES
Obs	3920	3906	3906
R^2^	0.8782	0.9635	0.8789

#### Non-linear feature test

**Fig 1:**
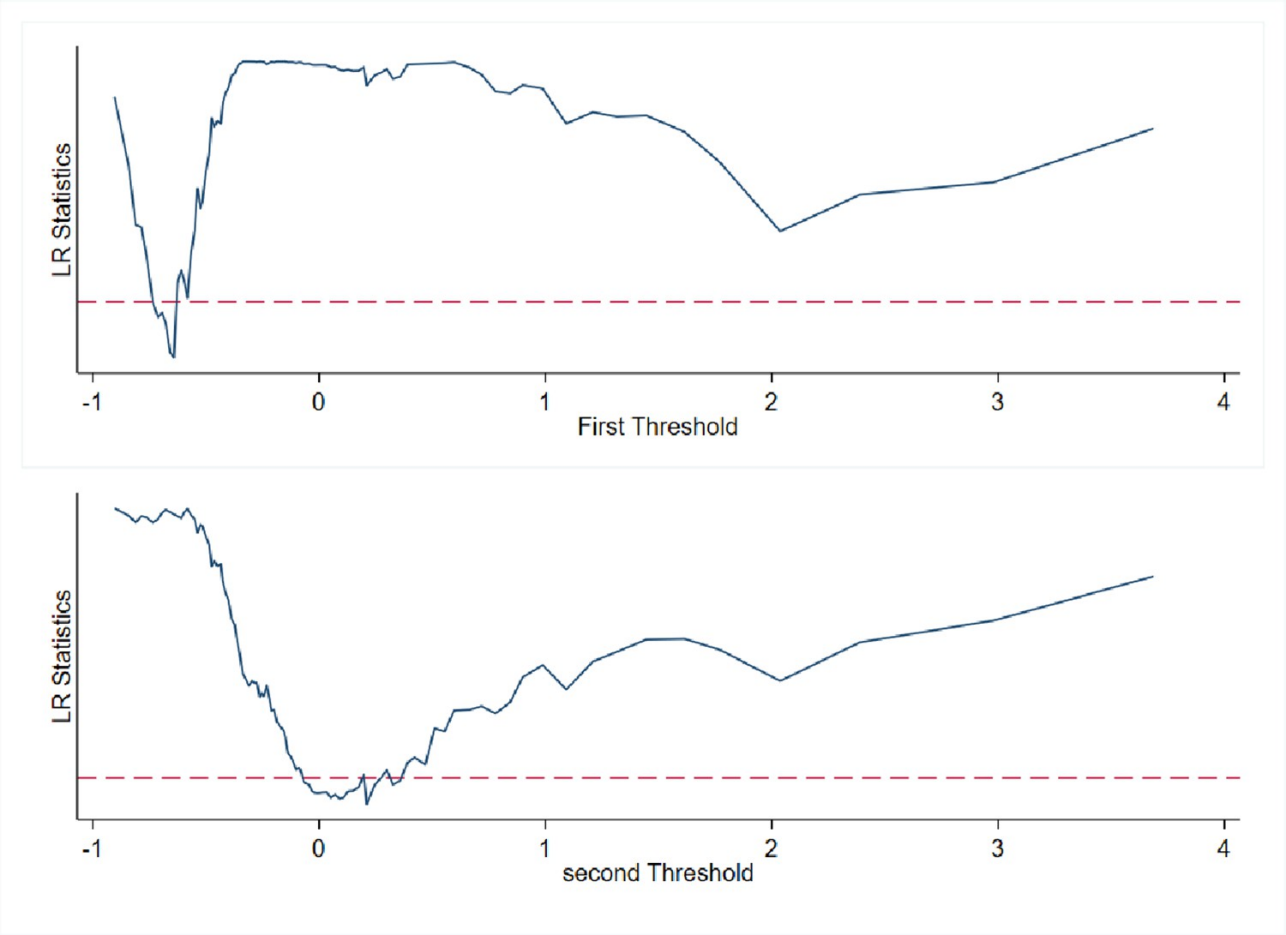
LR test chart.

To examine whether the promotion effect of DE on IS has non-linear characteristics, this study uses DE as the threshold variable and regresses based on the threshold model constructed above. Before performing threshold regression, it is essential to test the threshold model to determine the specific number of thresholds. The test results are shown in Table 7. Both the single threshold and double threshold pass the test, but the triple threshold test is not passed, indicating that the double threshold model should be chosen. Fig 1 presents the LR test chart for the double threshold, with threshold values of -0.6412 and 0.2114, respectively.

**Table 7:** Threshold test results.

Threshold variables	Threshold test	F value	P value	critical value		
				1%	5%	10%
de	Single threshold test	47.83**	0.0167	53.5193	39.4621	33.3633
	Double threshold test	50.36**	0.0233	57.5091	45.0291	38.4194
	Triple threshold test	15.83	0.5100	84.9708	69.5314	53.8362

Panel threshold regression results are presented in Table 8. When de is less than a certain value, the coefficient of de is 0.1723. When de falls between two specific values, the coefficient of de is 0.3428. When de is greater than a certain value, the coefficient of de is 0.0604. It is evident that the influence of DE on IS first increases and then decreases. When de is between specific values, the promotion effect of DE on IS level is the most substantial. This indicates that as DE continues to develop, its economic effect on IS becomes increasingly significant, making a more prominent positive impact on the upgrade of IS. However, when DE reaches a certain high level of development, its effect on IS will no longer be apparent. The discovery of the nonlinear effects reveals the intricate relationship between DE and IS. The observed inverted U-shaped trend, which first strengthens and then weakens, reflects the ’creative destruction’ process discussed in Schumpeter’s innovation theory (1934) [44]. In the early stages of DE, innovation drives rapid industrial upgrading; however, as time progresses, market saturation and the diminution of technology dividends may cause this impetus to wane.

**Table 8:** Threshold regression results.

	(1)
Variables	is
de (de≤−0.6412)	0.1723***
	(0.0318)
de (−0.6412<de≤0.2114)	0.3428***
	(0.0315)
de (de>0.2114)	0.0604***
	(0.0084)
Control	YES
Obs	3920
R^2^	0.4444

Our study provides empirical evidence for the ’creative destruction’ in the digital transformation process by revealing the nonlinear characteristics of DE’s impact on IS at different stages of development. This strengthening and subsequent weakening relationship, characterized by the inverted U-shaped curve, offers new insights for policymakers in devising strategies for the development of DE, particularly when considering how to balance the short-term shocks and long-term benefits of technological progress.

Artificial Intelligence (AI) has emerged as a vital catalyst initiating a new wave of technological revolution and industrial transformation. Technologies like machine learning and deep learning have accelerated the digital transformation, promoting the application of new technologies across various industries, allowing industries to adapt more quickly to the demands of DE. Davenport and Ronanki (2018) argue that AI helps improve production precision [45], while Zhang et al. (2017) emphasize the value of AI in innovation and research and development [46]. Overall, AI provides direction for the transformation of IS in DE and aids in the optimization and upgrade of IS [47]. Hence, this research hypothesizes that DE’s positive impact on IS levels is more pronounced in regions with advanced AI development. To validate this hypothesis, the study conducts a grouped regression test. Specifically, the study uses the number of AI companies in each city as a proxy for AI development level (ai) in various regions, with larger values indicating higher levels of AI development. Based on this, the study constructs a dummy variable for AI development level (ai), which is set to 1 if ai exceeds the median for the province in that year and 0 otherwise. Grouped regression results are shown in Table 9. The coefficient of de is only significant in the first column, indicating that the positive effect of DE on IS level is more pronounced in regions with higher levels of AI development, confirming the above hypothesis. The findings of this paper are consistent with Arntz et al. (2016) ’s theory [47] that technological innovation promotes IS change.

**Table 9:** Grouped regression results.

	(1)	(2)
Variables	ic	ic
de	0.0312***	0.0012
	(0.0090)	(0.0142)
Control	YES	YES
City_FE	YES	YES
Year_FE	YES	YES
Obs	1909	1681
R^2^	0.8773	0.8521

## Discussion

### Main findings and their relationship with existing research

Firstly, this research identified that DE exerts a considerable positive effect on IS level. This aligns with Brynjolfsson and McAfee’s (2014) research [48], which posits that digital technology is becoming the core driving force in the modern economy, exerting profound effects on the overall economic structure. Although DE has a positive role in promoting the level of IS, this study suggests that its impact manifests non-linear characteristics, consistent with the views of Fu [37]. Their research discerned a marginal decreasing non-linear feature in the impact of DE on the level of IS. However, diverging from this, our study identifies an inverted U-shaped feature in the influence of DE on IS level, providing a fresh perspective on understanding the intricate relationship between technological progress and industrial structural changes. This reminds us that the influence of DE on IS isn’t limitless, and there may be a saturation point. Consistent with past studies, this research also believes that when DE reaches a certain scale and depth, its promotional effect will gradually weaken.

Secondly, the findings of this study corroborate the viewpoint of Acemoglu [24], suggesting that HC plays a bridging role in the relationship between DE and industrial structural changes. This underlines the importance of education and training in the digital age, resonating with the conclusions of Autor et al. (2003) [27]. This finding further emphasizes that in the digital age, it is not only the development of technology itself that has an impact on industrial structural changes, but also the cultivation and improvement of HC levels.

Moreover, the exploration of spatial spillover effects in this study offers a new perspective. DE not only exerts influence within a region but also manifests its strength across different regions. This is supported by Chen (2023) study [49]. In addition, this paper also finds that the IS itself also has significant spatial dependence. The above findings suggest that inter-regional cooperation and integration should be considered when promoting the development of DE and IS to achieve a wider range of benefits [50, 51]. Especially in the cities we studied, the development of DE is not merely an internal affair of a single city but has formed an interconnected network effect between cities. Therefore, future policymakers and planners need to focus more on cross-regional collaboration and establish mechanisms for the coordinated development of DE to foster the collective prosperity of urban agglomerations.

The progression of AI technology is considered a key driving force behind DE. Our study’s analysis supports this viewpoint, revealing that in regions with higher AI development levels, DE’s positive influence on IS is more pronounced. This aligns with Davenport and Ronanki’s (2018) perspective [45], which states that AI technologies like machine learning and deep learning play a crucial role in enhancing production precision and fostering innovation. Additionally, Zhang et al. (2023) highlighted the value of AI in R&D activities, further corroborating this study’s findings [12]. This provides a strong guidance for the formulation of future DE development strategy and the application of artificial intelligence technology, and provides a substantial reference for the optimization of IS and the sustainable development of DE.

In summary, our empirical results are not only statistically significant, but also meaningful in economic theory. These findings provide a new perspective for understanding how DE affects IS, and provide a theoretical basis for policymakers to formulate relevant policies.

### Research limitations and future research directions

First, this study mainly focuses on the impact of DE on the level of IS without delving into the underlying mechanisms. For instance, how DE affects innovation and competitiveness across different industries remains to be further investigated. Secondly, although this research detects a non-linear relationship between DE and IS level, where the exact "saturation" or "inflection" point lies still demands more empirical tests for validation. Lastly, due to data constraints, the study is based on Chinese city data. Future extensions to other countries or regions would be beneficial to verify the universality of the findings from this study. In summary, this research provides a fresh lens for understanding the relationship between DE and IS, but there are still many avenues left unexplored. It’s hoped that future studies will make more profound contributions in this realm.

## Conclusion

Synthesizing the empirical analysis of this study, we draw the following conclusions: DE has a significant positive impact on the upgrading of IS in Chinese cities. This finding not only provides new empirical support for endogenous growth theory but also establishes an empirical basis for understanding how digital technology, as a general-purpose technology, affects industrial development. In particular, DE plays a key role in promoting the growth of the service sector and the transformation of manufacturing, aligning with existing theoretical assertions about technological innovation and industrial development. Furthermore, DE promotes the optimization and upgrading of IS by elevating the level of HC. The discovery of this mediating effect lends new empirical support to endogenous growth theory, where technological progress not only acts directly on industrial transformation but also indirectly promotes economic growth and industrial upgrading by improving the skills and knowledge level of the workforce.

Our research also finds that the impact of DE on industrial upgrading exhibits significant spatial spillover effects, highlighting the importance of considering inter-regional cooperation in DE development strategies. Furthermore, the nonlinear relationship we revealed between the development of DE and industrial upgrading has important implications for understanding the policies that should be adopted at different stages of development. Further heterogeneity analysis found that in cities with more developed artificial intelligence technology, DE has a more significant positive effect on the upgrading of IS. This highlights the need to take regional development differences into account when formulating relevant policies.

### Recommendations and implications

Based on the findings of this study, the following recommendations are proposed. Firstly, there’s a need to further promote the development of DE. The government should continuously enhance policy support and investment in DE, expedite the construction of digital infrastructure, and ensure the application and promotion of digital technology across various fields. Secondly, the emphasis on HC construction is crucial. In the context of DE, a high level of HC becomes vital. Investments in education and training, especially in digital skills training, should be ramped up to ensure the labor market aligns with the demands of DE. Thirdly, promoting inter-regional cooperation is essential. Given the significant spatial spillover effects of DE on IS level, regions should collaborate and collectively push DE’s development for shared prosperity. Fourthly, policymakers should closely monitor the non-linear impacts of DE. They should recognize the developmental stages of DE and make adjustments based on its influence on IS level to ensure its consistent and stable role in optimizing IS. Fifthly, there’s a need to encourage R&D and the application of high-end technologies, especially AI technologies. The government should increase financial support for the R&D of these technologies and create an environment that fosters innovation. By implementing the above measures, there’s hope to further boost the development of DE, optimize IS, and realize high-quality economic development.
